# Clinical Factors Associated with Adherence to the Follow-Up Examination after Positive Fecal Occult Blood Test in National Colorectal Cancer Screening

**DOI:** 10.3390/jcm9010260

**Published:** 2020-01-18

**Authors:** Byung Chang Kim, Minjoo Kang, Eunjung Park, Jeong-Im Shim, Shinhee Kang, Jessie Lee, Ha Jin Tchoe, Kyeong Ae Kong, Duk Hwan Kim, Yu Jin Kim, Kui Son Choi, Chang Mo Moon

**Affiliations:** 1 Center for Colorectal Cancer, Center for Cancer Prevention and Detection, Cancer Epidemiology Branch, Research Institute and Hospital, National Cancer Center, Goyang-si, Gyeonggi-do 10408, Korea; mdzara@ncc.re.kr; 2 National Evidence-Based Healthcare Collaborating Agency (NECA), Seoul, Korea; minjoo@neca.re.kr (M.K.); ejpark@neca.re.kr (E.P.); jis425@neca.re.kr (J.-I.S.); shkang@neca.re.kr (S.K.); halee@neca.re.kr (J.L.); hajin@neca.re.kr (H.J.T.); 3 Department of Preventive Medicine, College of Medicine, Ewha Womans University, Seoul 03760, Korea; joy-well@hanmail.net; 4 Digestive Disease Center, Department of Internal Medicine, CHA University School of Medicine, Gyeonggi-do 11160, Korea; teires.d.kim@gmail.com; 5 Department of Internal Medicine, Hallym University College of Medicine, Gangwon-do 24252, Korea; KIMYJ0614@gmail.com; 6 Department of Cancer Control and Population Health, Graduate School of Cancer Science and Policy, National Cancer Center, 323 Ilsan-ro, Ilsandong-gu, Goyang-si, Gyeonggi-do 10408, Korea; 7 Department of Internal Medicine, College of Medicine, Ewha Womans University, 1071 Anyangcheon-ro, Yangcheon-gu, Seoul 07985, Korea; 8 Tissue Injury Defense Research Center, Ewha Womans University, Seoul 03760, Korea

**Keywords:** colorectal cancer, fecal occult blood test, follow-up examination, compliance

## Abstract

Background: The compliance with the follow-up examination after a positive fecal occult blood test (FOBT) is lower than expected. We aimed to evaluate the adherence rate to the follow-up examination in patients with a positive FOBT and to identify the clinical factors associated with this adherence. Methods: The study population comprised adults aged ≥50 years who participated in the National Cancer Screening Program for colorectal cancer (CRC) in 2013. Compliance was defined as undergoing follow-up examination within 1 year of a positive FOBT. Results: From 214,131 individuals with a positive FOBT, 120,911 (56.5%) were in the compliance group and 93,220 (43.5%) were in the non-compliance group. On multivariate analysis, good compliance was associated with men (odds ratio (OR) = 1.12, 95% confidence interval (CI) (1.09–1.15)), younger ages (70–79 years, OR = 2.19 (2.09–2.31); 60–69 years, OR = 3.29 (3.13–3.46); 50–59 years, OR = 3.57 (3.39–3.75) vs. >80 years), previous experience of CRC screening (a negative FOBT, OR = 1.18 (1.15–1.21); a positive FOBT, OR = 2.42 (2.31–2.54)), absent previous experience of colonoscopy or barium enema (OR = 2.06 (1.99–2.13)), higher economic income (quartile, 75%, OR = 1.14 (1.11–1.17); 100%, OR = 1.22 (1.19–1.25)), current smokers (OR = 1.12 (1.09–1.15)), alcohol intake (OR = 1.03 (1.01–1.05)), active physical activity (≥3 times/week, OR = 1.13 (1.11–1.15)), depression (OR = 1.11 (1.08–1.14)), and present comorbidities (Charlson Comorbidity Index, ≥1). Conclusion: This study identified clinical factors, namely, male, younger ages, prior experience of fecal test, absent history of colonoscopy or double-contrast barium enema (DCBE) within 5 years, and high socioeconomic status to be associated with good adherence to the follow-up examination after a positive FOBT.

## 1. Introduction

Colorectal cancer (CRC) is the third most common malignancy in men and the second in women worldwide [1]. CRC is also the third ranked cancer in both men and women in Korea [2]. Screening tests reportedly reduce CRC incidence and related mortality [3,4], and therefore, national screening guidelines have been recommended for average-risk persons in several countries [5,6]. The Asia Pacific Working Group on Colorectal Cancer has recommended guidelines for CRC screening tests, including a fecal occult blood test (FOBT), colonoscopy, and flexible sigmoidoscopy [7]. In 2004, the Korean government conducted a nationwide CRC screening for Medical Aid recipients and National Health Insurance (NHI) beneficiaries in the lower 50% income bracket as a part of the National Cancer Screening Program (NCSP) [8]. The NCSP for CRC covers the annual FOBT as the primary screening modality for men and women aged over 50 years. The NCSP also covers a follow-up examination with either double-contrast barium enema (DCBE) or colonoscopy [8]. Annual or biennial FOBTs reportedly decrease CRC mortality by 15–35% in average populations, based on several large studies [9]. Despite these effects, the only participants of FOBTs have survival benefits through the follow-up test, such as colonoscopy and DCBE. Therefore, CRC screening guidelines recommend that colonoscopy is the preferred modality to further examine the individuals with a positive FOBT [10]. 

Even though the CRC screening is covered under NCSP, the participation rate is lower in Korea than in other countries [11,12,13]. In addition, the participation rate has been lower for CRC screening than other cancers, such as stomach, breast, and cervix cancer; the participation rate was 25.65–32.9% from 2009 to 2012 in the Korean NCSP [14]. Additionally, the adherence to the follow-up examination after a positive FOBT was also poor, ranging from 42% to 86%, and few studies have evaluated the clinical factors that influence it [8,15,16,17,18,19,20,21]. The previous study, which underwent based on the data of the NCSP in Korea, missed the participants who performed follow-up tests (colonoscopy or DBCE) outside the NCSP because the data of NCSP did not include the tests conducted through the National Health Insurance Service (NHIS) or private cancer screening clinics and didn’t analyze the association the clinico-behavioral factors with comorbidities and the adherence [8]. There is a need to identify the clinical characteristics of individuals who did not take a follow-up test and to eventually find a way to improve the participation of individuals with a FOBT (+) in the follow-up test as a part of the NCSP according to these factors based on the NCSP and NHIS. Therefore, in the present study, we aimed to evaluate the actual adherence rate to the follow-up examination in individuals with a positive FOBT based on the NCSP and NHIS and to identify the clinical factors associated with this adherence in the Korean NCSP. 

## 2. Materials and Methods

### 2.1. Data Source and Study Cohort

**Figure 1:**
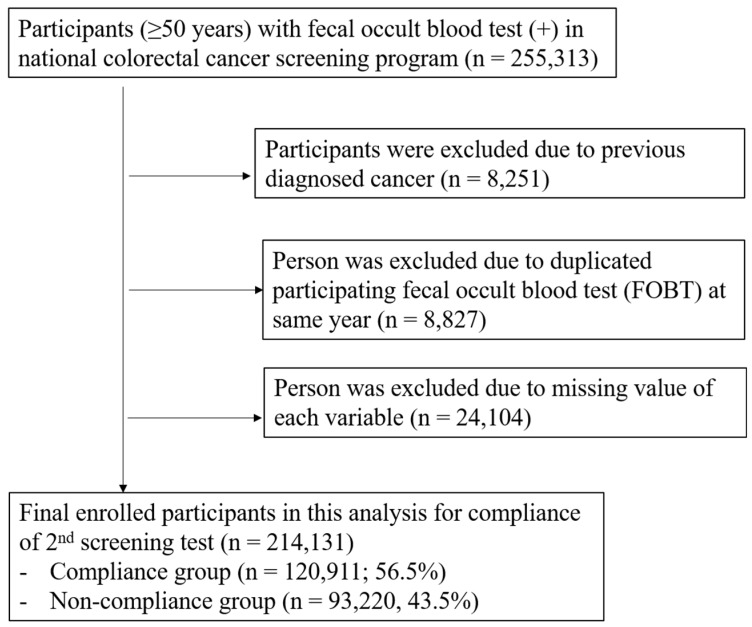
Flow diagram of the study population.

We linked NCSP data and claims data from the Korea National Health Insurance Service database (KNHIS, NHIS-2017-1-352) to establish our study population. (Supplementary Figure S1) The study population was defined as adults ≥50 years with a positive FOBT in the NCSP. Regarding the adherence rate of the follow-up examination after a positive FOBT, we included individuals who participated in the NCSP for CRC from 2009 to 2013. In order to identify clinical factors related to adherence to the follow-up examination, we included individuals from 1 January to 31 December 2013. The number of participants with a FOBT (+) was 255,313 in 2013. The participants who had been previously diagnosed with CRC (*n* = 8251; 3.2%) and those with a duplicated FOBT in 2013 (*n* = 8827; 3.5%) were excluded. The participants with missing values for each variable were excluded (*n* = 24,104). The final number of enrolled participants was 214,131. Among them, 120,911 (56.5%) underwent the follow-up examination within 1 year, whereas 93,220 (43.5%) did not appear for the follow-up test within 1 year of the FOBT (+) result. The study subjects are summarized in Figure 1. 

### 2.2. Definition

Individuals who underwent the follow-up examination (colonoscopy or barium enema) within 1 year of a positive FOBT result were defined as the compliance group, whereas those who did not undergo the follow-up test within 1 year were allocated to the non-compliance group. To investigate the status of follow-up tests after positive FOBTs, we used the data from the results of a colonoscopy or DCBE in the NCSP and KNHIS health insurance claims database. Korea offers universal access to health care, regardless of ability to pay, through the National Health Insurance (NHI) and Medical Aid Program (MAP). Every Korean resident is universally eligible for NHI and is required to pay health insurance contributions except for those supported by the MAP. The NHI covers 97% of the population, while the remaining 3% are covered by the MAP. NHI insured are classified into two groups; NHI (company) represented the people (employee) hired by the company and NHI (local) represented the people hired by themselves (self-employed). Health insurance premium levels (based on the quartile) were calculated based on the annual insurance fee, which was assessed by people’s annual income and property. Quartile 25% represented the lower 25% of the lower health insurance premium group in health insurance premium level. Quartile 50% stood for the range from 25% to 50% of the lower health insurance premium. Quartile 75% stood for the range from 50% to 75% of the high health insurance premium. Quartile 100% stood for the range from 75% to 100% of the high health insurance premium. Their groups represented their economic status.

The clinical and socioeconomic information was acquired from the KNHIS (NHIS-2017-1-352) and NCSP database, and the variables included in the study were selected based on previous studies [8,15,22]. Demographic data, various clinical factors (e.g., height, weight, blood pressure, blood glucose level, total cholesterol, triglyceride, high-density lipoprotein (HDL), low-density lipoprotein (LDL), previous past history of diseases, family history of diseases, consumption of alcohols, smoking status, and physical activity), FOBT results, and the follow-up examination (colonoscopy and DCBE) findings after FOBT screening were obtained from the KNHIS database and Korean NCSP. Using the NHIS claims database, we could confirm whether people with FOBT positives had received a therapeutic or diagnostic DCBE or colonoscopy test (Figure S1). The relevant claims codes we considered were as follows: HA032 (DCBE), E7660 (colonoscopy), E7670 (rectoscopy), E7680 (sigmoidoscopy), Q7720 (polypectomy under sigmoidoscopy), Q7752 (endoscopic mucosal resection under sigmoidoscopy), Q7701 (endoscopic polypectomy under colonoscopy), Q7703 (endoscopic mucosal resection under colonoscopy), QX703 (partial endoscopic submucosal dissection), and QX706 (endoscopic submucosal dissection). The previous history of colonoscopy or DCBE was obtained from the KNHIS and NCSP database for the last 5 years.

The information regarding comorbidities (e.g., the Charlson Comorbidity Index (CCI), anemia, hemorrhagic, and hematologic diseases, etc.) were obtained from the KNHIS database through the International Classification of Diseases 10th Revision (ICD-10) code (Table S1). Our study was approved by the National Evidence-Based Healthcare Collaborating Agency Institutional Review Board (NECAIRB 17-005). We analyzed the data based on the definition of old age (aged 65 or more) in Korea. 

### 2.3. Statistical Analysis

In baseline characteristics of the study population, the statistical comparison of proportions among groups was performed by the Chi-square test in univariate analysis. Clinical factors with *p* values <0.05 in univariate analysis were subsequently estimated with odds ratios (ORs) and 95% confidence intervals (CIs) using multivariate logistic regression analysis. Model 1 was analyzed for factors associated with the compliance with the follow-up test by adjusting for each variable. Model 2 was estimated with ORs by adjusting for all variables using logistic regression analysis. All statistical analyses were carried out using SAS statistical software. 

## 3. Results 

### 3.1. Demographic and Clinical Factors 

The adherence rate of the follow-up test after the FOBT (+) was almost similar every year at about 58% from 2009 to 2013 (Table S2). About 45–50% of the subjects with a positive FOBT underwent the follow-up test through the NCSP, while another 10–13% underwent the follow-up test through medical services by the KNHIS.

Baseline characteristics of the study population according to the compliance with the follow-up test after a positive FOBT in 2013 are shown in Table 1. The demographic parameters identified to be associated with adherence to the follow-up test were male sex, younger ages (50–59 years), and ages in the sixties (60–69 years). Previous experience of colonoscopy or DCBE, a FOBT within 1 year, and higher payment of health insurance were more frequently observed in the compliance group than in the non-compliance group. Most study subjects were insured by occupational NHI, and the order of medical facilities that received the FOBT were private clinics, hospitals, and general hospitals in both groups, respectively. In addition, comorbid diseases (CCI ≥1), osteoporosis, and depression were positively related to compliance with the follow-up test. The life-style factors associated with good compliance were high body mass index (BMI) (≥25 kg/m^2^), alcohol intake, and high physical activity (≥3 times/week). The residence area, anemia, and smoking were not significantly associated with the compliance to the follow-up examination (Table 1). 

### 3.2. Odds Ratio of Compliance Factors for the Follow-Up Test after a Positive FOBT

We analyzed the factors and ORs related to additional screening after the FOBT (+) in the NCSP and NHIS data using the logistic regression models (Models 1 and 2) (Table 2). In Model 2, men attended the additional study more than women (OR = 1.12, *p* < 0.0001), and younger groups also gradually increased their ORs of attending the follow-up test compared with the older aged group (>80 aged) after a FOBT (+) (70–79 years, OR = 2.19; 60–69 years, OR = 3.29; 50–59 years, OR = 3.57). Many medical factors were associated with increasing the compliance with the follow-up additional test: No previous experience of colonoscopy or DCBE (OR = 2.06, *p* < 0.0001), history of undergoing a FOBT within 1 year (negative FOBT, OR = 1.18; positive FOBT, OR = 2.42 vs. no experience), high economic status (quartile, 75%, OR = 1.14; 100%, OR = 1.22), history of the use of specific medical facilities within 6 months before the FOBT (general hospital, OR = 1.14; hospital, OR = 1.46 vs. clinic), and local or occupational NHI comparing to the MAP (OR = 1.44 and 1.38). Disease-related factors were also associated with increased follow-up adherence after the FOBT (+): Presence of comorbidities (CCI ≥1), absence of hematologic and hemorrhagic diseases, and presence of osteoporosis and depression. Some life-style factors, such as alcohol intake (OR = 1.03) and smoking (OR = 1.12) were associated with high compliance with the follow-up additional test, while high physical activity (≥3 times/week) was also related to this outcome (OR = 1.13). However, high BMI (≥25 kg/m^2^) did not affect the compliance in Model 2 and was not related with compliance of the FOBT in Model 1, either. (Table 2).

### 3.3. Factors Associated with Adherence to the Follow-Up Test after a Positive FOBT According to Age

We investigated the related factors of the follow-up screening test after a FOBT (+) in the NCSP using subgroup analysis based on age. On stratified analysis with age, male sex, absent personal history of colonoscopy or DCBE, pre-existing history of a FOBT within 1 year, recent history of the use of medical facilities, and high economic status (>50% quartile of health insurance premium level) were related to compliance with the follow-up screening test in both age groups. The experience of a positive FOBT within 1 year was highly associated with greater adherence to the follow-up test after the FOBT (+) of the NCSP than an experience of a negative FOBT or no experience of a fecal test in both age groups. The medical facilities related with the hospital (general hospital and hospital) were related to high compliance with the follow-up test, but some medical facilities were less compliant for the follow-up test than other medical facilities. 

Physical activity, smoking, and alcohol intake were also associated with adherence to the additional follow-up test in both age groups. The presence of comorbidities (CCI ≥1), osteoporosis, and depression also positively affected the follow-up screening test adherence regardless of the age group. Anemia and BMI were not associated with additional screening test adherence in the young age group in Model 2. The type of health insurance and the presence of hemorrhagic and hematologic diseases did not affect the follow-up screening test adherence after a FOBT (+) in the old age group.

NHI comparing with the MAP increased the compliance with the follow-up test in the young age group but it did not affect the result in the old age group. Alcohol intake also increased compliance in the young age group but decreased it in the old age group. However, in the urban area, there was a decreased compliance in the young age group but it increased in the old age group (Table 3).

## 4. Discussion

In the current study, 120,911 (56.5%) participants underwent the follow-up test within 1 year, whereas 93,220 (43.5%) did not receive the follow-up test within 1 year of the FOBT (+) result. The adherence rate of the follow-up test after the FOBT (+) was almost similar every year at about 58% from 2009 to 2013 (Table S2). The follow-up rate was low despite the fact that the Korean NCSP provided a free follow-up test for the participants with a positive FOBT. 

There may be many reasons to explain why only half of the participants underwent the recommended follow-up test. One of possible explanations for the low follow-up is that the results of the FOBT were delivered to the participants by mailed letters in the NCSP of Korea. It may have been more difficult for the elderly to interpret the FOBT results and understand the need to undergo the follow-up with DCBE or colonoscopy. A reminder system is needed for improving the adherence. Further, the health care system may also have had an impact on the low follow-up rate. Previous studies showed that a general practitioner or family doctor played a major role in explaining the entirety of the screening process (follow-up test, etc.) and providing the test [16,18]. However, in the NCSP, a FOBT is performed at a clinic or hospital that has been designated as a CRC screening unit, and those who are invited to undergo screening can visit any of these certified CRC screening units. The lack of a systematic referral system or continuous connection between a physician in the CRC screening unit and participants, which does exist between a family doctor and their patients, might have affected the low follow-up rate. Further, absence of a systematic call and recall system and family doctor system may also be associated with the low compliance rate of other types of cancer screening in the NCSP.

However, the follow-up rate in the current study may have been under-estimated because it did not take into account the cases undergoing the follow-up test privately not through the NCSP or KNHIS because the information of private health facility examinations is not incorporated in national data. In Korea, colonoscopy or DCBE tests are widely conducted in outpatient clinics or private screening centers. Therefore, if the follow-up conducted in the private sector were included, we would expect the follow-up rate to increase.

The demographic factors associated with the follow-up examination were male sex and younger individuals. The compliance with the follow-up test after the FOBT (+) was higher in the younger group in this study. These results were in agreement with those of other previous studies reporting relatively high adherence in younger individuals than in the elderly and increased proportion of the male sex in the follow-up test [8]. In the NCSP, there is no upper age limit for CRC screening. Therefore, the lower compliance with the follow-up test among individuals aged 70 or over affected the overall low follow-up rate.

Participants who had previously undergone a CRC screening test were associated with adherence to the follow-up additional test after the FOBT (+). In the present study, individuals with no experience of colonoscopy or DCBE within the last 5 years and a FOBT within the last 1 year showed higher adherence rates than persons with such prior experiences. These results were in agreement with those of other previous studies. Correia et al. also reported that individuals with a recent prior colonoscopy experience (particularly within 2 years) are less likely to appear for a follow-up colon examination [23]. The participants who recently underwent a colon examination with colonoscopy or DCBE did not feel the need to appear for a follow-up test after the FOBT (+) because doctors had already explained their colonoscopy or DCBE surveillance schedule. However, the adherence rate of individuals with a FOBT (+) was much higher than those of persons with a negative FOBT in multivariate analysis (a positive FOBT, OR = 2.42; a negative FOBT, OR = 1.18, respectively). 

Socioeconomic status was also related with the follow-up test after the FOBT (+). These results were consistent with those of previously reported studies, that CRC screening use varies according to age and income [11,12]. In the current study, NHI (local or occupational) beneficiaries adhered to the follow-up additional test more often than Medical Aid recipients, even though the follow-up test was free in the NCSP. In addition, the persons with high payment from the NHI (>50% quantile) were more likely to undertake the follow-up test than those with lower payment after the FOBT (+). This result is also in agreement with those of other previous studies [8]. However, the NCSP provides free-of-charge screening services and follow-up testing and finical support for those who participated in the NCSP and were subsequently diagnosed with CRC through the program. Thus, financial cost directly related to cancer screening was not a major obstacle of adherence to the follow-up test. This observation can be explained by the fact that Medical Aid recipients were less concerned about the expected benefits from the additional test [8]. Medical Aid recipients and low-income NHI beneficiaries were also predominantly older individuals, and they were mostly unemployed and economically inactive. Therefore, they were more likely to face barriers with benefitting from hospital facilities, which reflected as low participation of Medical Aid recipients and low economic NHI beneficiaries of the NCSP in the present study [24]. Further, some individuals who received the result of the FOBT (+) by mail occasionally were not able to completely understand the results; these were primarily the individuals with low socioeconomic status and with low economic levels and literacy. The NCSP should prepare a supporting system for easy access to the follow-up test for patients with Medical Aid recipients and for economically vulnerable groups.

A few comorbidities of the screened individuals were associated with the follow-up screening test after a positive FOBT in the present study. The presence of comorbidities (CCI ≥ 1), absence of hematologic and hemorrhagic diseases, osteoporosis, and depression were predictors of high compliance with the follow-up test after the FOBT (+) in the current study, based on nationwide data. Contrary to the current data, the presence of one or two comorbidities was a predictor of failure to the follow-up with colonoscopy after the FOBT (+); however, this result is in comparison with patients having many comorbidities (≥8 comorbidities) [23]. Carlson et al. reported using Carlson score that shows that comorbidity was not associated with appearing for the follow-up screening test [21]. The discrepancy may have been caused by the difference between enrolled patients of the current study (≥50 years old) and Carlson’s study (≥70 years old). 

The lifestyle of screening a person was also associated with a follow-up CRC screening test. The predictors of high adherence to the follow-up were high physical activity (≥3 times/week), alcohol consumption, and smoking in the present study. These factors are also related with the lifestyle, and individuals with healthy lifestyle might have been more concerned about the information on health risks like CRC, consequently making them more willing to undergo CRC screening and risk assessments [25,26]. Conversely, lack of CRC risk perception was usually associated with unhealthy lifestyles, such as smoking or obesity, and also with low economic status [26]. 

The limitations of the current study can be identified from our results. This study did not analyze the physician’s effect on the follow-up test after the FOBT (+) in the NCSP. The subsequent examinations after the FOBT (+) were affected by the physician’s recommendation in several studies [16,27]. The results of the FOBT in the Korean NCSP were noted for the individuals informed by mailing service who attended the screening program. Therefore, the physician’s effect on the follow-up test may not be significant in the present study. This study couldn’t identify the participants who underwent the colonoscopy in private health check-up facilities after a positive FOBT. The data of private health facilities are not shared with the KNHIS or NCSP. Therefore, the adherence rate might be underestimated in this study. The other limitation was data inaccuracy in that some data that were gathered through subjective questionnaire surveys of the NCSP (e.g., physical activity, smoking, and alcohol) and requested bill codes for co-morbid diseases of the NHIS. These data were influenced by the subjective propensity of individuals who appeared for the screening test and doctors’ subjective tendencies of requesting a bill to the NHIS.

The present findings have several strengths. The analysis was based on a nationwide population-based cohort, including NCSP and NHIS data. The current data showed precise and expansive information of the individuals who attended the national CRC screening program. The present results revealed the associated factors that could predict higher compliance with the follow-up test. Further, we analyzed the effects of comorbidities on complete colon examination among individuals who appeared for the follow-up test after the FOBT (+). This data gave us clues for improving the compliance with the follow-up colon examination for patients with comorbidities.

## 5. Conclusions

In conclusion, we identified the factors associated with adherence to the follow-up test after a positive FOBT in the NCSP. Male sex, young aged individuals, and high socioeconomic status were identified as the predictors of good adherence. In terms of previous experience of CRC screening, previous history of colonoscopy or DCBE was negatively associated with good adherence, while previous history of FOBTs was positively associated with adherence. Participants with comorbid diseases also had high performance rates of appearing for the follow-up test after the FOBT (+). To further improve the attendance, a support system or policy should be considered based on these associated factors. 

## Figures and Tables

**Table 1:** Baseline characteristics of individuals according to the compliance to the follow-up test after a positive FOBT in 2013.

	Total *N* = 214,131	Non-Compliance Group *N* = 93,220		Compliance Group *N* = 120,911		*p*-Value
	*N*	*N*	(%)	*N*	(%)	
Sex						
Men	112,465	48,261	(42.9)	64,204	(57.1)	<0.0001
Women	101,666	44,959	(44.2)	56,707	(55.8)	
Age, years						
50–59	86,466	34,628	(40.0)	51,838	(60.0)	<0.0001
60–69	72,177	29,281	(40.6)	42,896	(59.4)	
70–79	46,942	23,427	(49.9)	23,515	(50.1)	
≥80	8546	5884	(68.9)	2662	(31.1)	
Previous experience of colonoscopy or DCBE within 5 years						
Yes	194,788	88,064	(45.2)	106,724	(54.8)	<0.0001
No	19,343	5156	(26.7)	14,187	(73.3)	
Previous experience of a FOBT within 1 year						
Negative	179,623	80,664	(49.2)	98,959	(50.8)	<0.0001
Positive	24,441	10,226	(41.8)	14,215	(58.2)	
No experience	10,067	2330	(23.1)	7737	(76.9)	
History of medical facility use (within 6 months before and after a FOBT)						
General Hospital	33,894	13,913	(41.0)	19,981	(59.0)	<0.0001
Hospital	41,493	16,017	(38.6)	25,476	(61.4)	
Clinic	133,023	59,980	(45.1)	73,043	(54.9)	
Others *	5721	3310	(57.9)	2411	(42.1)	
Type of Health Insurance **						
NHI (local)	63,692	26,897	(42.2)	36,795	(57.8)	<0.0001
NHI (company)	144,277	63,262	(43.8)	81,015	(56.2)	
MAP	6162	3061	(49.7)	3101	(50.3)	
Residence area						
Urban area ***	86,293	37,449	(43.4)	48,844	(56.6)	0.2949
Rural area †	127,838	55,771	(43.6)	72,067	(56.4)	
Health insurance premium level (based on the quartile) ‡						
Quartile 25%	50,761	23,504	(46.3)	27,257	(53.7)	<0.0001
Quartile 50%	46,357	20,570	(44.4)	25,787	(55.6)	
Quartile 75%	49,381	20,669	(41.9)	28,712	(58.1)	
Quartile 100%	67,632	28,477	(42.1)	39,155	(57.9)	
Types of medical facility that performed a FOBT						
General Hospital	47,236	19,988	(42.3)	27,248	(57.7)	<0.0001
Hospital	67,078	29,393	(43.8)	37,685	(56.2)	
Clinic	99,643	43,737	(43.9)	55,906	(56.1)	
Others *	174	102	(58.6)	72	(41.4)	
CCI						
0	79,457	35,318	(44.4)	44,139	(55.6)	<0.0001
1	65,016	28,220	(43.4)	36,796	(56.6)	
2	39,895	16,868	(42.3)	23,027	(57.7)	
3	29,763	12,814	(43.1)	16,949	(56.9)	
Anemia						
Absent	186,172	80,997	(43.5)	105,175	(56.5)	0.5069
Present	27,959	12,223	(43.7)	15,736	(56.3)	
Hemorrhagic and hematologic diseases						
Absent	210,124	91,414	(43.5)	118,710	(56.5)	0.0476
Present	4007	1806	(45.1)	2201	(54.9)	
Osteoporosis						
Absent	172,796	74,847	(43.3)	97,949	(56.7)	<0.0001
Present	41,335	18,373	(44.4)	22,962	(55.6)	
Depression						
Absent	181,782	79,538	(43.8)	102,244	(56.2)	<0.0001
Present	32,349	13,682	(42.3)	18,667	(57.7)	
BMI (kg/m^2^)						
<25	134,107	58,972	(44.0)	75,135	(56.0)	<0.0001
≥25	80,024	34,248	(42.8)	45,776	(57.2)	
Alcohol intake						
Absent	134,894	59,517	(44.1)	75,377	(55.9)	<0.0001
Present	79,237	33,703	(42.5)	45,534	(57.5)	
Smoking						
Absent	136,636	59,438	(43.5)	77,198	(56.5)	0.6815
Present	77,495	33,782	(43.6)	43,713	(56.4)	
Physical activity						
<3 times/week	79,729	36,675	(46.0)	43,054	(54.0)	<0.0001
≥3 times/week	134,402	56,545	(42.1)	77,857	(57.9)	

* Nursing home, public health center, etc. ** The National Health Insurance (NHI) insured are classified into two groups; NHI (company) represented the people (employees) hired by the company and NHI (local) represented the people hired by themselves (self-employed). *** Urban area was defined as the capital and metropolitan cities in Korea (Seoul, Busan, Daegu, Incheon, Gwangju, Daejeon, and Ulsan). † Rural area was defined as the provinces in Korea (Gyeonggi, Gangwon, Chungbuk, Chungnam, Jeonbuk, Jeonnam, Gyeongbuk, Gyeongnam, and Jeju). ‡ Health insurance premium levels (based on the quartile) were calculated based on the annual insurance fee, which was assessed by people’s annual income and property. The quartiles 25, 50, 75, and 100% represented their paid insurance fee levels in an ascending manner. Their groups represented the economic status. Fecal occult blood test (FOBT); double contrast barium enema (DCBE); National Health Insurance (NHI); Medical Aid Program (MAP); Charlson comorbidity index (CCI); body mass index (BMI).

**Table 2:** Clinical factors associated with adherence to the follow-up test after a positive FOBT by multivariable analysis.

Variables	Model 1			Model 2		
	OR	(95% CI)	*p*-Value	OR	(95% CI)	*p*-Value
Gender						
Men	1.06	(1.04–1.07)	<0.0001	1.12	(1.09–1.15)	<0.0001
Women	1.00			1.00		
Age						
50–59 years	3.31	(3.15–3.47)	<0.0001	3.57	(3.39–3.75)	<0.0001
60–69 years	3.24	(3.09–3.40)	<0.0001	3.29	(3.13–3.46)	<0.0001
70–79 years	2.22	(2.11–2.33)	<0.0001	2.19	(2.09–2.31)	<0.0001
≥80 years	1.00			1.00		
Previous experience of colonoscopy or DCBE within 5 years						
Yes	1.00			1.00		
No	2.27	(2.20–2.35)	<0.0001	2.06	(1.99–2.13)	<0.0001
Previous experience of a FOBT within 1 year						
Negative	1.13	(1.10–1.16)	<0.0001	1.18	(1.15–1.21)	<0.0001
Positive	2.71	(2.58–2.84)	<0.0001	2.42	(2.31–2.54)	<0.0001
No experience	1.00			1.00		
History of medical facility used (within 6 months before and after a FOBT)						
Clinic	1.00			1.00		
General Hospital	1.18	(1.15–1.21)	<0.0001	1.14	(1.11–1.17)	<0.0001
Hospital	1.31	(1.28–1.34)	<0.0001	1.46	(1.42–1.50)	<0.0001
Others *	0.60	(0.57–0.63)	<0.0001	0.71	(0.67–0.75)	<0.0001
Type of Health Insurance **						
MAP	1.00			1.00		
NHI (company)	1.26	(1.20–1.33)	<0.0001	1.38	(1.31–1.46)	<0.0001
NHI (local)	1.35	(1.28–1.42)	<0.0001	1.44	(1.36–1.53)	<0.0001
Residence area						
Rural area ***	1.00			1.00		
Urban area †	1.01	(0.99–1.03)	0.29	1.00	(0.98–1.02)	0.82
Health insurance premium level (based on the quartile) ‡						
Quartile 25%	1.00			1.00		
Quartile 50%	1.08	(1.05–1.11)	<0.0001	1.02	(0.99–1.04)	0.29
Quartile 75%	1.20	(1.17–1.23)	<0.0001	1.14	(1.11–1.17)	<0.0001
Quartile 100%	1.19	(1.16–1.21)	<0.0001	1.22	(1.19–1.25)	<0.0001
Types of medical facility that performed a FOBT						
Clinic	1.00			1.00		
General Hospital	1.07	(1.04–1.09)	<0.0001	1.02	(0.99–1.04)	0.21
Hospital	1.00	(0.98–1.02)	0.76	0.82	(0.80–0.84)	<0.0001
Others *	0.55	(0.41–0.75)	0.0001	0.72	(0.53–0.98)	0.04
CCI						
0	1.00			1.00		
1	1.04	(1.02–1.07)	<0.0001	1.08	(1.06–1.11)	<0.0001
2	1.09	(1.07–1.12)	<0.0001	1.16	(1.13–1.19)	<0.0001
3	1.06	(1.03–1.09)	<0.0001	1.16	(1.12–1.19)	<0.0001
Anemia						
Absent	1.00			1.00		
Present	0.99	(0.97–1.02)	0.51	1.00	(0.98–1.03)	0.75
Hemorrhagic and hematologic diseases						
Absent	1.00			1.00		
Present	0.94	(0.88–1.00)	0.05	0.93	(0.87–0.99)	0.03
Osteoporosis						
Absent	1.00			1.00		
Present	0.96	(0.94–0.98)	<0.0001	1.09	(1.06–1.11)	<0.0001
Depression						
Absent	1.00			1.00		
Present	1.06	(1.04–1.09)	<0.0001	1.11	(1.08–1.14)	<0.0001
BMI (kg/m^2^)						
<25	1.00			1.00		
≥25	1.05	(1.03–1.07)	<0.0001	1.01	(0.99–1.03)	0.45
Alcohol intake						
Absent	1.00			1.00		
Present	1.07	(1.05–1.09)	<0.0001	1.03	(1.01–1.05)	0.01
Smoking						
Absent	1.00			1.00		
Present	1.00	(0.98–1.01)	0.68	1.12	(1.09–1.15)	<0.0001
Physical activity						
<3 times/week	1.00			1.00		
≥3 times/week	1.17	(1.15–1.19)	<0.0001	1.13	(1.11–1.15)	<0.0001

* Nursing home, public health center, etc. ** The NHI insured are classified into two groups; NHI (company) represented the people (employees) hired by the company and NHI (local) represented the people hired by themselves (self-employed). *** Rural area was defined as the provinces in Korea (Gyeonggi, Gangwon, Chungbuk, Chungnam, Jeonbuk, Jeonnam, Gyeongbuk, Gyeongnam, and Jeju). † Urban area was defined as the capital and metropolitan cities in Korea (Seoul, Busan, Daegu, Incheon, Gwangju, Daejeon, and Ulsan). ‡ Health insurance premium levels (based on the quartile) were calculated based on the annual insurance fee, which was assessed by people’s annual income and property. The quartiles 25, 50, 75, and 100% represented their paid insurance fee levels in an ascending manner. Their groups represented the economic status. Model 1 was analyzed for factors associated with the compliance with the follow-up test by adjusting for each variable. Model 2 was estimated with odds ratios (ORs) by adjusting for all variables using multivariate logistic regression analysis. Odds ratio (OR); confidence interval (CI); fecal occult blood test (FOBT); double contrast barium enema (DCBE); National Health Insurance (NHI); Medical Aid Program (MAP); Charlson comorbidity index (CCI); body mass index (BMI).

**Table 3:** Clinical factors associated with adherence to the follow-up test after a positive FOBT, depending on the age group (65 years old).

Variables	<65 Years Old						≥65 Years Old					
	Model 1			Model 2			Model 1			Model 2		
	OR	(95% CI)	*p*-Value	OR	(95% CI)	*p*-Value	OR	95% CI	*p*-Value	OR	95% CI	*p*-Value
Gender												
Men	0.96	(0.94–0.98)	0.0003	1.04	(1.01–1.07)	<0.0001	1.21	(1.18–1.24)	<0.0001	1.20	(1.15–1.24)	<0.0001
Women	1.00			1.00			1.00			1.00		
Previous experience of colonoscopy or DCBE within 5 years												
Yes	1.00			1.00			1.00			1.00		
No	2.18	(2.09–2.28)	<0.0001	1.98	(1.89–2.07)	0.02	2.45	(2.33–2.57)	<0.0001	2.26	(2.15–2.37)	<0.0001
Previous experience of a FOBT within 1 year												
Negative	1.07	(1.03–1.11)	0.0005	1.10	(1.06–1.14)	<0.0001	1.27	(1.22–1.33)	<0.0001	1.28	(1.23–1.34)	<0.0001
Positive	3.22	(3.02–3.44)	<0.0001	2.97	(2.78–3.18)	<0.0001	2.18	(2.03–2.34)	<0.0001	1.85	(1.72–1.99)	<0.0001
No experience	1.00			1.00			1.00			1.00		
History of medical facility used (within 6 months before and after a FOBT)												
Clinic	1.00			1.00			1.00			1.00		
General Hospital	1.17	(1.13–1.20)	<0.0001	1.17	(1.13–1.21)	<0.0001	1.18	(1.14–1.23)	<0.0001	1.11	(1.06–1.16)	<0.0001
Hospital	1.32	(1.28–1.36)	<0.0001	1.47	(1.41–1.52)	<0.0001	1.20	(1.15–1.24)	<0.0001	1.44	(1.18–1.51)	<0.0001
Others *	0.67	(0.61–0.73)	<0.0001	0.70	(0.63–0.76)	<0.0001	0.63	(0.59–0.68)	<0.0001	0.68	(0.63–0.73)	<0.0001
Type of Health Insurance **												
MAP	1.00			1.00			1.00					
NHI (company)	1.49	(1.40–1.58)	<0.0001	1.48	(1.39–1.58)	<0.0001	0.98	(0.89–1.09)	0.73	0.92	(0.83–1.02)	0.24
NHI (local)	1.63	(1.53–1.73)	<0.0001	1.57	(1.46–1.67)	<0.0001	0.98	(0.89–1.09)	0.73	0.94	(0.84–1.04)	0.12
Residence area												
Rural area ***	1.00			1.00			1.00			1.00		
Urban area †	0.96	(0.94–0.98)	0.0002	0.97	(0.95–0.99)	0.01	1.07	(1.04–1.09)	<0.0001	1.05	(1.02–1.08)	0.001
Health insurance premium level (based on the quartile) ‡												
Quartile 25%	1.00			1.00			1.00			1.00		
Quartile 50%	1.05	(1.02–1.09)	0.002	1.01	(0.98–1.05)	0.48	1.06	(1.02–1.11)	0.004	1.05	(1.00–1.09)	0.04
Quartile 75%	1.21	(1.17–1.25)	<0.0001	1.15	(1.11–1.19)	<0.0001	1.14	(1.09–1.18)	<0.0001	1.14	(1.09–1.18)	<0.0001
Quartile 100%	1.29	(1.25–1.33)	<0.0001	1.22	(1.17–1.26)	<0.0001	1.17	(1.13–1.21)	<0.0001	1.17	(1.13–1.22)	<0.0001
Types of medical facility that performed a FOBT												
Clinic	1.00			1.00			1.00			1.00		
General Hospital	1.03	(1.00–1.06)	0.03	0.97	(0.94–1.00)	0.07	1.11	(1.07–1.15)	<0.0001	1.09	(1.05–1.13)	<0.0001
Hospital	1.06	(1.03–1.09)	<0.0001	0.86	(0.84–0.89)	<0.0001	0.90	(0.87–0.93)	<0.0001	0.77	(0.74–0.80)	<0.0001
Others *	0.52	(0.33–0.81)	0.004	0.60	(0.38–0.94)	0.03	0.62	(0.41–0.94)	0.02	0.85	(0.56–1.29)	0.45
CCI												
0	1.00			1.00			1.00			1.00		
1	1.11	(1.08–1.14)	<0.0001	1.10	(1.07–1.13)	<0.0001	1.05	(1.01–1.08)	0.01	1.03	(1.00–1.07)	0.07
2	1.19	(1.15–1.22)	<0.0001	1.17	(1.13–1.21)	<0.0001	1.13	(1.09–1.18)	<0.0001	1.10	(1.06–1.15)	<0.0001
3	1.17	(1.12–1.22)	<0.0001	1.14	(1.10–1.19)	<0.0001	1.15	(1.10–1.19)	<0.0001	1.10	(1.05–1.14)	<0.0001
Anemia												
Absent	1.00			1.00			1.00			1.00		
Present	1.07	(1.03–1.11)	0.0003	1.02	(0.98–1.06)	0.31	0.99	(0.95–1.02)	0.45	0.96	(0.93–1.00)	0.06
Hemorrhagic and Hematologic diseases												
Absent	1.00			1.00			1.00			1.00		
Present	0.96	(0.88–1.06)	0.43	0.91	(0.83–1.00)	0.04	0.99	(0.90–1.08)	0.73	0.94	(0.86–1.03)	0.1536
Osteoporosis												
Absent	1.00			1.00			1.00			1.00		
Present	1.17	(1.13–1.21)	<0.0001	1.12	(1.07–1.16)	<0.0001	0.98	(0.95–1.01)	0.19	1.07	(1.04–1.11)	<0.0001
Depression												
Absent	1.00			1.00			1.00			1.00		
Present	1.11	(1.07–1.15)	<0.0001	1.08	(1.04–1.12)	<0.0001	1.11	(1.07–1.15)	<0.0001	1.11	(1.07–1.15)	<0.0001
BMI (kg/m^2^)												
<25	1.00			1.00			1.00			1.00		
≥25	1.01	(0.99–1.04)	0.30	0.99	(0.97–1.02)	0.63	1.09	(1.06–1.12)	<0.0001	1.09	(1.06–1.13)	<0.0001
Alcohol intake												
Absent	1.00			1.00			1.00			1.00		
Present	1.06	(1.04–1.09)	<0.0001	1.03	(1.00–1.06)	0.02	0.88	(0.86–0.91)	<.0001	0.96	(0.93–0.99)	0.01
Smoking												
Absent	1.00			1.00			1.00			1.00		
Present	1.11	(1.09–1.14)	<0.0001	1.12	(1.09–1.16)	<0.0001	0.92	(0.89–0.95)	<0.0001	1.05	(1.02–1.09)	0.004
Physical activity												
<3 times/week	1.00			1.00			1.00			1.00		
≥3 times/week	1.08	(1.06–1.11)	<0.0001	1.09	(1.06–1.11)	<0.0001	1.29	(1.26–1.33)	<0.0001	1.25	(1.22–1.29)	<0.0001

* Nursing home, public health center, etc. ** The NHI insured are classified into two groups: NHI (company) represented the people (employees) hired by the company and NHI (local) represented the people hired by themselves (self-employed). *** Rural area was defined as the provinces in Korea (Gyeonggi, Gangwon, Chungbuk, Chungnam, Jeonbuk, Jeonnam, Gyeongbuk, Gyeongnam, and Jeju). † Urban area was defined as the capital and metropolitan cities in Korea (Seoul, Busan, Daegu, Incheon, Gwangju, Daejeon, and Ulsan). ‡ Health insurance premium levels (based on the quartile) were calculated based on the annual insurance fee, which was assessed by people’s annual income and property. The quartiles 25, 50, 75, and 100% represented their paid insurance fee levels in an ascending manner. Their groups represented the economic status. Model 1 was analyzed for factors associated with the compliance with the follow-up test by adjusting for each variable. Model 2 was estimated with ORs by adjusting for all variables using multivariate logistic regression analysis. Odds ratio (OR); confidence interval (CI); fecal occult blood test (FOBT); double contrast barium enema (DCBE); National Health Insurance (NHI); Medical Aid Program (MAP); Charlson comorbidity index (CCI); body mass index (BMI).

## Supplementary Materials

The supplementary materials are available online at https://www.mdpi.com/2077-0383/9/1/260/s1.
