# Supplementary material for: Clinical Factors Associated with Adherence to the Follow-Up Examination after Positive Fecal Occult Blood Test in National Colorectal Cancer Screening
Source: J Clin Med. 2020 Jan 18;9(1):260. doi: 10.3390/jcm9010260 (PMC7019756; doi:10.3390/jcm9010260)
Supplement: Supplementary file 1 [file jcm-09-00260-s001.pdf]

Table S1. Clinical conditions in ICD-10 (International Classification of Diseases 10th Revision) code

**Clinical condition**	**ICD-10 code**	
Charlson Comorbidity Index (CCI)		Weight
Myocardial Infarction	I21, I22, I252	1
Congestive Heart Failure	I43, I50, I099, I110, I130, I132, I255, I420, I425, I426, I427, I428, I429, P290	1
Peripheral Vascular Disease	I70, I71, I731, I738, I739, I771, I790, I792, K551, K558, K559, Z958, Z959	1
Cerebrovascular Disease	G45, G46, I60, I61, I62, I63, I64, I65, I66, I67, I68, I69, H340	1
Dementia	F00, F01, F02, F03, G30, F051, G311	1
Chronic Pulmonary Disease	J40, J41, J42, J43, J44, J45, J46, J47, J60, J61, J62, J63, J64, J65, J66, J67, I278, I279, J684, J701, J703	1
Connective Tissue Disease-Rheumatic Disease	M05, M32, M33, M34, M06, M315, M351, M353, M360	1
Peptic Ulcer Disease	K25, K26, K27, K28	1
Mild Liver Disease	B18, K73, K74, K700, K701, K702, K703, K709, K717, K713, K714, K715, K760, K762, K763, K764, K768, K769, Z944	1
Diabetes without complications	E100, E101, E106, E108, E109, E110, E111, E116, E118, E119, E120, E121, E126, E128, E129, E130, E131, E136, E138, E139, E140, E141, E146, E148, E149	1
Diabetes with complications	E102, E103, E104, E105, E107, E112, E113, E114, E115, E117, E122, E123, E124, E125, E127, E132, E133, E134, E135, E137, E142, E143, E144, E145, E147	2
Paraplegia and Hemiplegia	G81, G82, G041, G114, G801, G802, G830, G831, G832, G833, G834, G839	2
Renal Disease	N18, N19, N052, N053, N054, N055, N056, N057, N250, I120, I131, N032, N033, N034, N035, N036, N037, Z490, Z491, Z492, Z940, Z992	2
Cancer	C00, C01, C02, C03, C04, C05, C06, C07, C08, C09, C10, C11, C12, C13, C14, C15, C16, C17, C18, C19, C20, C21, C22, C23, C24, C25, C26, C30, C31, C32, C33, C34, C37, C38, C39, C40, C41, C43, C45, C46, C47, C48, C49, C50, C51, C52, C53, C54, C55, C56, C57, C58, C60, C61, C62, C63, C64, C65, C66, C67, C68, C69, C70, C71, C72, C73, C74, C75, C76, C81, C82, C83, C84, C85, C88, C90, C91, C92, C93, C94, C95, C96, C97	2
Moderate or Severe Liver Disease	K704, K711, K721, K729, K765, K766, K767, I850, I859, I864, I982	3
Metastatic Carcinoma	C77, C78, C79, C80	3
AIDS/HIV	B20, B21, B22, B24	6

Anemia	D50-59, D60-64
Hemorrhagic & Hematologic diseases	D65–D69, D70-D77
Osteoporosis	M80-M82
Depression	F06.32, F31.3, F31.4, F32, F33, F341, F38.1, F41.2

Table S2. Adherence rate of follow-up examination after FOBT (+) from 2009 to 2013

	**2009**		**2010**		**2011**		**2012**		**2013**	
	**N=143,344**		**N=180,662**		**N=195,249**		**N=232,831**		**N=255,313**	
	**N**	**(%)**	**N**	**(%)**	**N**	**(%)**	**N**	**(%)**	**N**	**(%)**
Compliance group										
within NCSP	64,943	(45.3)	80,853	(44.8)	89,238	(45.7)	116,388	(50.0)	123,018	(48.2)
within KNHIS	18,535	(12.9)	23,789	(13.2)	24,090	(12.3)	25,943	(11.1)	26,821	(10.5)
Non-compliance group	59,866	(41.8)	76,020	(42.1)	81,921	(42.0)	90,500	(38.9)	105,474	(41.3)

NCSP, Korea National Cancer Screening Program; KNHIS, Korea National Health Insurance Service.
